# Bedside Sonographic Duplex Technique as a Monitoring Tool in Patients after Decompressive Craniectomy: A Single Centre Experience

**DOI:** 10.3390/medicina56020085

**Published:** 2020-02-19

**Authors:** Habib Bendella, Joachim Spreer, Alexander Hartmann, Alhadi Igressa, Marc Maegele, Rolf Lefering, Makoto Nakamura

**Affiliations:** 1 Department of Neurosurgery, University of Witten/Herdecke, Cologne Merheim Medical Center (CMMC), 51109 Cologne, Germany; HartmannA@kliniken-koeln.de (A.H.); Igressa@yahoo.com (A.I.); NakamuraM@kliniken-koeln.de (M.N.); 2 Division of Neuroradiology, Department of Radiology, University of Witten/Herdecke, Cologne Merheim Medical Center (CMMC), 51109 Cologne, Germany; SpreerJ@kliniken-koeln.de; 3 Department of Traumatology, Orthopedic Surgery and Sportsmedicine, University of Witten/Herdecke, Cologne-Merheim Medical Center (CMMC), 51109 Cologne, Germany; Marc.Maegele@t-online.de; 4 Institute for Research in Operative Medicine (IFOM), University of Witten/Herdecke, Cologne-Merheim, 51109 Cologne, Germany; rolf.lefering@uni-wh.de

**Keywords:** basal cerebral arteries, brain trauma, cranial computed tomography, decompressive craniectomy, ultrasound, ventricle dimensions

## Abstract

*Background and objectives:* Bedside sonographic duplex technique (SDT) may be used as an adjunct to cranial computed tomography (CCT) to monitor brain-injured patients after decompressive craniectomy (DC). The present study aimed to assess the value of SDT in repeated measurements of ventricle dimensions in patients after DC by comparing both techniques. *Materials and Methods:* Retrospective assessment of 20 consecutive patients after DC for refractory intracranial pressure (ICP) increase following subarachnoid hemorrhage (SAH), bleeding and trauma which were examined by SDT and CCT in the context of routine clinical practice. Whenever a repeated CCT was clinically indicated SDT examinations were performed within 24 hours and correlated via measurement of the dimensions of all four cerebral ventricles. Basal cerebral arteries including pathologies such as vasospasms were also evaluated in comparison to selected digital subtraction angiography (DSA). *Results:* Repeated measurements of all four ventricle diameters showed high correlation between CCT and SDT (right lateral r = 0.997, *p* < 0.001; left lateral r = 0.997, *p* < 0.001; third r = 0.991, *p* < 0.001, fourth ventricle r = 0.977, *p* < 0.001). SDT performed well in visualizing basal cerebral arteries including pathologies (e.g., vasospasms) as compared to DSA. *Conclusions:* Repeated SDT measurements of the dimensions of all four ventricles in patients after DC for refractory ICP increase delivered reproducible results comparable to CCT. SDT may be considered as a valuable bedside monitoring tool in patients after DC.

## 1. Introduction

Decompressive craniectomy (DC) in patients after traumatic brain injury (TBI) with increased refractory intracranial pressure (ICP) may result in lower mortality as compared to patients with only medical management [1]. To monitor these critically ill patients for potential complications after DC, e.g., tissue swelling and alterations in ventricular dimensions, cranial computed tomography (CCT) is considered as the “gold standard” [2]. However, CCT often requires the transport of a critically ill patient from the intensive care unit (ICU) to the CCT scanner and vice versa which may expose the patient to additional and potentially harmful risks [3,4,5,6]. Significant alterations in ICP have been documented during in-hospital transports, transfer onto the CCT table and during CCT scanning where elevated head-positioning could no longer be maintained [4,7]. Even a brief increase in ICP with a corresponding decrease in cerebral perfusion pressure (CPP) may harm the patient and lead to secondary brain ischemia [8,9]. To avoid these “second hits” which may substantially impact patient outcomes, in-hospital transports should be restricted to a minimum. 

Recently, the sonographic duplex technique (SDT) has been suggested as an adjunct to CCT to diagnose and monitor TBI patients after DC [10,11,12]. The sonographic duplex technique has been introduced as a reliable bedside tool that is available in almost every ICU and is already being used by a number of centers to monitor DC patients. However, its reproducibility within a single individual on a time scale has not yet been demonstrated to prove the reliability of the method. It is further assumed that SDT may also be used to assess cerebral basal arteries and to visualize potential pathologies such as the occurrence of functional stenoses due to vasospasms which have strongly been associated with neurological deterioration and delayed cerebral ischemia [13,14,15,16]. The “gold standard” in this context is still considered invasive digital subtraction angiography (DSA) [17]. The aim of the present study was to assess the clinical value of repeated SDT for estimating ventricle diameters in identical patients after DC for refractory ICP increase via intra-individual comparison with CCT. 

## 2. Materials and Methods

We retrospectively assessed 20 consecutive patients (11 males and 9 females; mean age 56.9 ± 17.6 years) who had undergone DC between April 2014 and September 2016 for different pathologies and at least three control CCTs within their further sequelae whenever clinically indicated. The indication to perform a follow-up CCT was driven by a persistent increase in ICP and/or clinically neuro worsening. The time span between the three measurements was variable. Out of the 20 patients, 4 patients (20%) had suffered from subarachnoid hemorrhage (SAH), 3 (15%) from intracranial bleedings and 13 (65%) from traumatic brain injuries (TBI). Due to elevated ICP as assessed via imaging or ICP measurement DC was performed on the pathological hemisphere: in 10 (50%) cases on the right, in 6 (30%) cases on the left and in 4 (20%) cases on both hemispheres.

SDT after CCT was performed on the same day of CCT by three independent examiners and measurements from both methods were compared for all four ventricle dimensions. Patients that had undergone any other specific or unspecific intervention between CCT and SDT were excluded as a priori from the study. Standard SDT assessment also included the evaluation of the basal cerebral arteries, both supratentorial and infratentorial. In cases in which functional stenoses had been detected via SDT, DSA was additionally performed for comparison. While control CCTs were part of routine clinical monitoring in the sequelae after DC, non-invasive SDT has been reported with the absence of any clinical side effects [18] and is widely acceptanced as a routine clinical assessment tool in a wide spectrum of disciplines. All assessments were conducted in the context of clinical routine practice, no prospective protocol was applied, and all results were reported in a descriptive manner. For analysis, all data were processed anonymously. The retrospective study design and the study protocol were approved by the Central Ethics Committee of the University Witten/Herdecke (UW/H CEC approval # 57/2019, from 21.05.2019).

### 2.1. CCT and SDT Imaging

CCT was performed by using a Somatom Definition Flash (Siemens, Forchheim, Germany). A spiral scan was acquired (100 kV, single collimation width 0.6 mm; pitch 0.55, matrix 512 x 512) and reformatted in contiguous 5 mm slices in the axial plane, and angulated according to a line connecting the anterior and posterior commissures, and in the coronal plane.

For the cerebral angiograms, a biplanar flat panel angiography (Philips Allura, Best, The Netherlands) was used. In patients with suspected stenoses in SDT, angiographic images in at least two different planes were acquired. Stenoses were detected visually and confirmed via DSA.

SDT was performed by using Hitachi Sono MR EUB 7500 and Hitachi Aloka Arietta V70 (Hitachi, Tokyo, Japan) with a Convex probe 5–2 (mechanical index 0.8 to 1.2) [18]. We placed the probe with sterile contact gel on the skin parallel for the axial plane and for the coronal plane at an angle of 45 degrees to the orbitomeatal line of the craniectomized side with a varying angle between probe surface and skin to visualize the ventricular system and the supra- and infratentorial basal cerebral arteries [18]. To assess the ventricular dimensions, the diameters of the lateral, the third and fourth ventricles were measured visually in each CCT and SDT. Repeated measurements in each patient were compared. The diameter was defined as the widest point orthogonal to the tangent of the outer ventricular wall, the plane of the midline, and the septum pellucidum. For the measurements, the sonographic plane in SDT is matched to the CCT slice in the axial and coronal plane. All examinations and measurements in both, SDT and CCT, were performed blinded to the results of the other method. If clinically indicated SDT was done in patients with stenoses after SAH before DSA. The previously measured values in SDT were compared to the results in DSA.

All measurements of the ventricular dimensions in the CCT and the basal cerebral arteries were done blinded to the values of SDT.

### 2.2. Statistical Analysis

Statistical analyses were completed using Microsoft Excel (version 2007 Microsoft Corp., Redmond, USA) and SPSS (version 22, IBM Corp., Armonk, NY, USA). Data are presented as mean ± standard deviation (SD). A descriptive analysis of the three consecutive time points was performed. The associations between the different measurements were analyzed using Pearson’s correlation coefficient r. The mean difference of CCT-SDT measurement was calculated with 95% confidence interval (CI) for each ventricle dimension. In order to analyze the agreement between the two methods, a Bland-Altman plot was generated. In addition, we provided a linear regression analysis. A *p*-value below 0.05 was considered statistically significant. The number of 60 pairs of measurement (3 time points in 20 individual cases) per location was chosen in order to calculate 95% confidence intervals of mean values with a precision of ± 0.25 x SD.

## 3. Results

The basic demographics of the patients included in this study are summarized in Table 1. 

### 3.1. Ventricle Assessment

**Figure 1:**
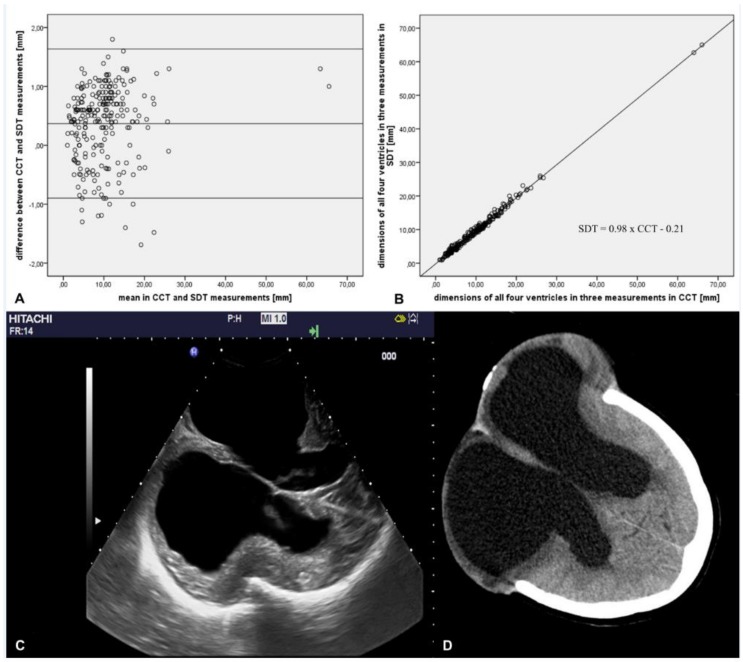
(**A**–**D**) Comparison of sonographic duplex technique (SDT) and cranial computed tomography (CCT). (**A**) Bland Altman test of ventricular size in SDT and CCT (n = 240). (**B**) Correlation of dimensions of all four ventricles in three measurements in SDT (y-axis) and CCT (x-axis). Linear correlation between the ventricular dimension in SDT and CCT is shown. The formula of the linear regression is SDT = 0.98 x CCT−0.21. Exemplar axial slices for both techniques ((**C**) SDT and (**D**) CCT).

**Figure 2:**
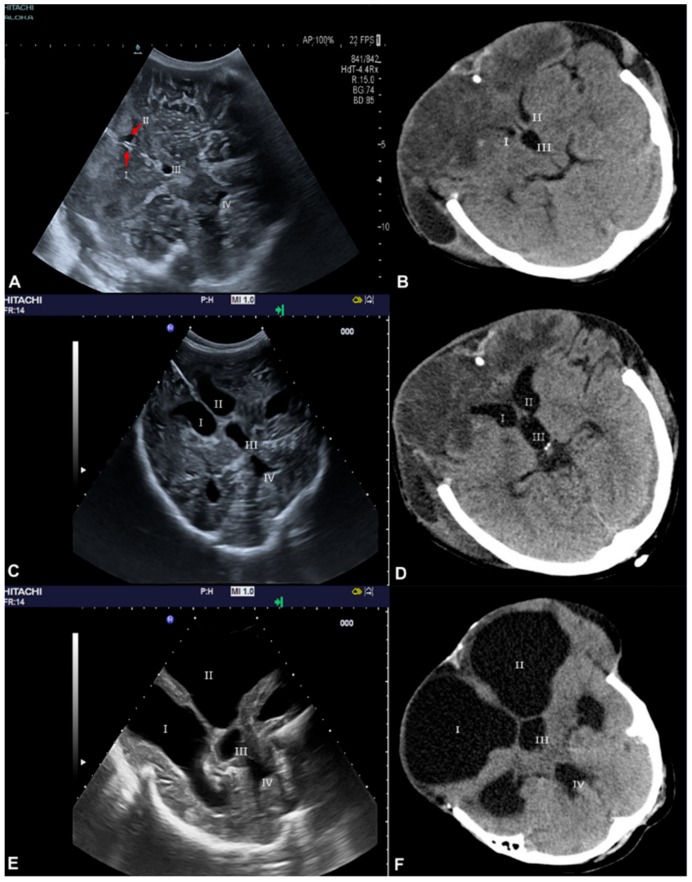
(**A**–**F**) Serial SDTs (**A**,**C**,**E**) and CCT (**B**,**D**,**F**) in a patient after craniectomy. All four small ventricles detected by SDT (**A**) could be demonstrated within one single plane compared to CCT (**B**). The dimensions of all four ventricles increased during the second and third measurements detected by SDT in one single plane (**C**,**E**) and compared with CCT (**D**,**F**).

Table 2 provides an overview of both SDT and CCT measurements of all four ventricles including comparisons. The mean difference between CCT-SDT measurement was 0.2–0.5 mm. There was a high correlation between both methods for all four ventricles with all comparisons reaching significance (*p* < 0.001). Figure 1A indicates no systematic bias observed in the mean difference between the two methods for all four ventricles and for the three measurements. Figure 1B shows a high correlation between values obtained by the two methods for all four ventricles for the three measurements (Pearson’s Correlation Coefficient r for all three measurement with *p* < 0.001: right lateral ventricle r = 0.997, left lateral ventricle r = 0.997, third ventricle r = 0.991, fourth ventricle r = 0.977). The right and lateral ventricles were visualized in both SDT (c) and CCT (d). An exemplar visualization of all four ventricles via both methods during the sequential measurements at the three different time points with the confirmation of ventricular enlargement over time is shown in Figure 2 (SDT in Figure 2A,C,E, and CCT Figure 2B,D,F).

### 3.2. Cerebral Arteries

**Figure 3:**
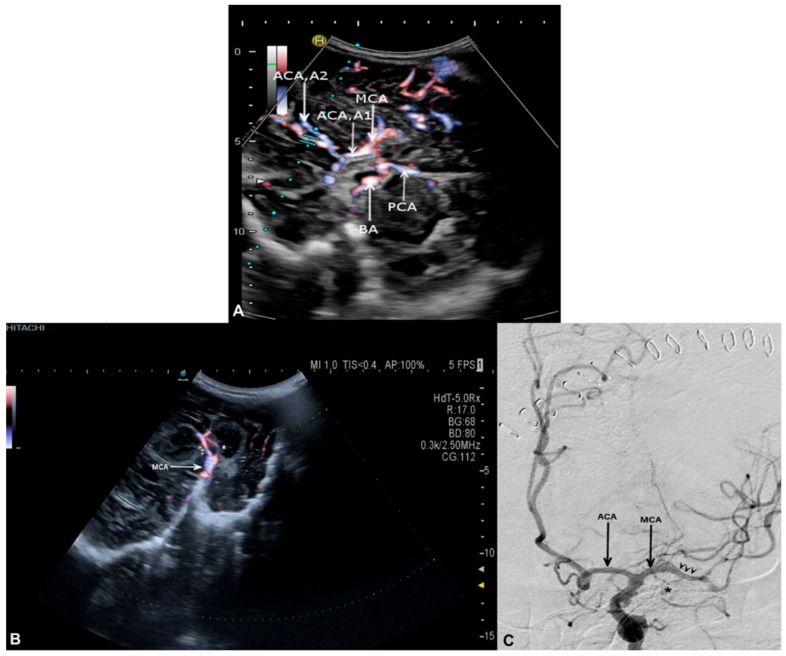
(**A**–**C**) Visualization of Willis circle and stenoses of the middle cerebral artery. SDT in the axial plane (**A**) in a patient after hemicraniectomy showing the Willis circle including the proximal (A1) and distal (A2) segment of the anterior cerebral artery (ACA), middle cerebral artery (MCA), posterior cerebral artery (PCA) and basilar artery (BA). SDT in the coronal plane (**B**) and DSA (**C**) in a patient after DC following SAH with stenoses of the middle cerebral artery (MCA) during vasospasm (arrowhead). Little branches (*) could also be shown by SDT (**B**) as well as by DSA (**C**).

**Figure 4:**
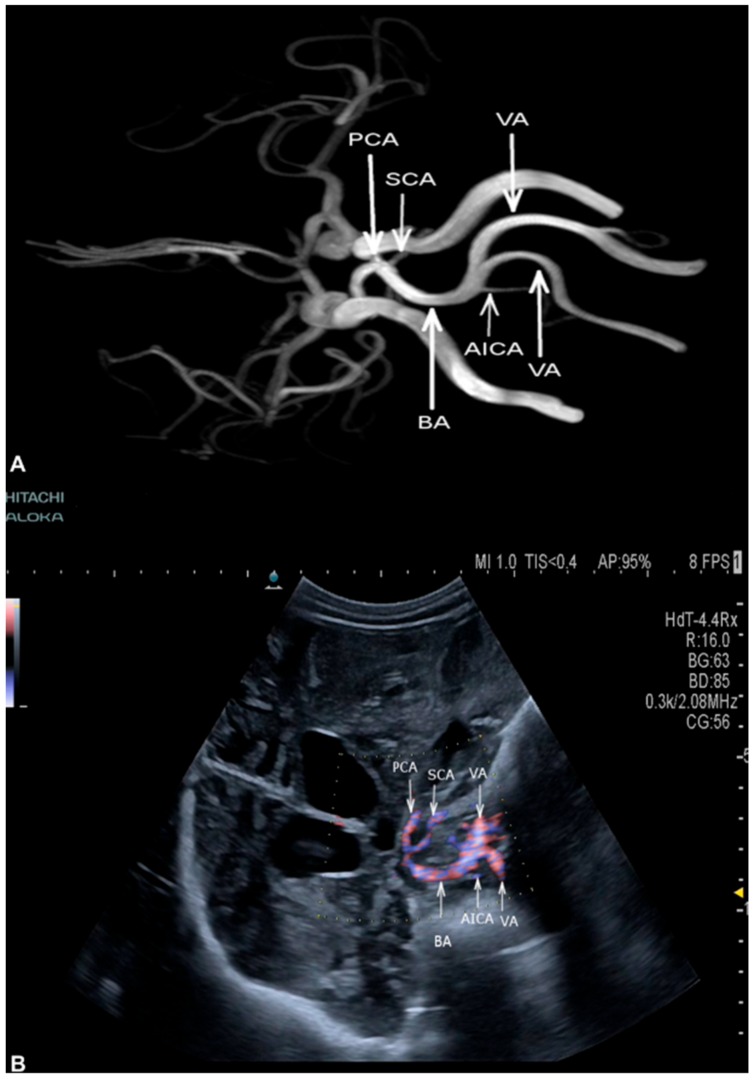
(**A**,**B**) Visualization of Willis circle is demonstrated via MR-angiography (**A**) and SDT (**A**). SDT in the coronal plane detects all branches of the infratentorial part of the Willis circle with vertebral arteries (VA), basilar artery (BA), anterior inferior cerebellar artery (AICA), superior cerebellar artery (SCA) and posterior cerebral artery (PCA).

The visualization of the basal supra- and infratentorial arteries via SDT was achieved in all cases. In three of the four cases with SAH as underlying pathology, functional stenoses due to vasospasms were visualized. As illustrated in Figure 3 and Figure 4, all cerebral arteries were assessed, both supra- and infratentorial, through SDT. Both anterior cerebral arteries (ACA) including both the proximal (A1) and distal (A2) segment are shown in Figure 3A. Stenoses due to vasospasms of the middle cerebral artery (MCA) as well as small branches of the vessel were detected by SDT in the coronal plane (Figure 3B) in comparison to DSA (Figure 3C). MR-angiography (Figure 4A) was used to illustrate all basal cerebral arteries especially within the infratentorial aspect, marked by a circle. The infratentorial cerebral arteries were obtained by SDT; Figure 4B shows the assessment of both vertebral arteries (VA), basilar artery (BA), anterior inferior cerebellar artery (AICA), superior cerebellar artery (SCA) and posterior cerebral artery (PCA) by SDT.

## 4. Discussion

Decompressive craniectomy in TBI with refractory ICP increase has been shown to reduce mortality but was associated with poor neurological outcomes, such as severe disability or vegetative state [1]. Therefore, continuous monitoring of these patients to enable early detection to avoid ongoing or further neurological deterioration is of utmost importance. Currently, these patients typically undergo CCT as the “gold standard” for assessment and monitoring. However, CCT in patients after DC may be associated with considerable risks in unstable patients, for example during in-hospital transports to the CCT scanner. It has been shown, that the transfer onto the CT table and the supine body positioning during the examination may potentially increase ICP which may lead to critical drops in CPP resulting in secondary cerebral ischemia [3,4,5,6,7,8]. A bedside monitoring method for follow-up during further neurocritical care after DC on the ICU is thus desirable. As shown recently, bedside SDT may have the potential to serve as an adjunct to CCT in the assessment of ventricular dimensions with a high correlation to the “gold standard” [10,11,12,19]. In the present study, serial quantitative measurements of all four ventricle dimensions in identical patients after DC via SDT were compared to CCT with intraclass correlation coefficients ranging between 0.977 and 0.997 for all three measurements over time. From these results, SDT may be considered as a reliable method to assess ventricular dimensions. This underpins the clinical significance and the value of a bedside monitoring technique to assess temporal changes in ventricle dimensions to a similar degree as the “gold standard”, which is currently still the CCT, thereby prompting timely intervention and avoiding poor outcomes in patients after DC. 

SDT is considered as a non-invasive bedside tool to monitor critically ill patients after DC and is almost universally available. It is clear that SDT cannot replace CCT in general and may rather serve as an adjunct to it. For example, if regular SDT indicates the progression of ventricle diameters but without a corresponding increase in ICP and/or neurological deterioration, no universal action may be prompted. However, regardless of SDT findings, increased ICP and/or neurological deteriorations would always prompt a CCT to assess the underlying cause. Likewise, the inter-rater variability would be high and a CCT would be needed if the SDT signal is found to be suspicious. Thus, SDT and CCT may not be counted as interchangeable but complementary. One major limitation of the present study was that the time interval between CCT and subsequent SDT was not consistently documented throughout the study. In general, SDT was performed after CCT on the same day of CCT and all efforts were undertaken to keep this interval as short as possible.

To date, there has not been any clinically relevant side effect yet reported from the use of ultrasonography [18]. The thermal index of the given procedure is considered rather low according to the British Medical Ultrasound Guideline 2009 [18]. SDT may be employed to quantify and follow-up ventricular enlargements. The findings of the present study show that the method may be suitable for routine clinical use. Over the past decade, there has been a consistent improvement of the ultrasound technology since it was first described in 1989 as a bedside technique to assess craniectomized patients through the transtemporal window [20,21,22]. Further development of the technology led to the clinical application of SDT, which allowed the correct spatial imaging of intracranial arteries [23,24]. As previously shown, SDT as a bedside tool may be considered as a reliable method and tool for the identification and differentiation of specific brain structures including pathologies, such as hematomas, midline shifts, ventricular enlargements as well as probe or drain positioning [10,11,12]. 

In addition, SDT was expanded to examine cerebral arteries both supra- and infratentorial in patients with SAH. The early diagnosis of stenoses due to vasospasms following SAH may support the indication for timely treatment in order to prevent cerebral ischemia and to improve neurological outcomes [25]. Vasospasms are difficult to detect based upon neurological symptoms especially in patients with significant neurological impairment at baseline. Stenoses due to vasospasms may lead to an increase in vascular resistance with consecutively reduced cerebral blood flow hereby potentially inducing secondary brain ischemia [26,27]. In the present study, stenoses of the cerebral arteries could be visualized via SDT in three out of four SAH patients during the vasospastic interval. We were able to visualize stenoses of the middle cerebral artery (MCA) which was confirmed by DSA, the “gold standard” for the diagnosis of stenoses due to vasospasms. In this context, Transcranial Doppler (TCD) which has been shown as a means to assess differences in blood flow velocity, only provides indirect proof for the presence of vasospasms [28,29]. Thus, SDT may also represent a reliable real-time and non-invasive imaging tool capable to visualize stenoses due to vasospasms. However, this technique has its limitations, for example in detecting and monitoring ischemic lesions [10]. The utility of SDT in the assessment of cerebral blood vessels is limited to anatomical stenosis; TCDs performed at bedside may have the potential to provide information related to ongoing vasospasms. These problems, however, may be overcome in the future through advances in image resolution and quality. Of note, the quality of the assessment may always remain with the investigator and is experience-dependent. 

## 5. Conclusions

We conclude, that SDT may serve as a complementary non-invasive bedside tool to identify individual patients with impending neurological failure with the advantage of reduced radiation exposure. The repeated measurements of the ventricular dimensions of all four ventricles in the same patient by bedside SDT is highly correlated with CCT in patients after DC. SDT was suitable for routine clinical use to detect ventricle diameters. We were able to visualize all supra- and infratentorial cerebral arteries with SDT. Therefore, SDT may be an option to visualize stenoses due to vasospasms and to initiate further diagnostic and timely treatment but these findings need further experimental and clinical validation.

## Figures and Tables

**Table 1:** Patient demographics and characteristics.

Characteristics	
N	20
Age (years)	56.9 ± 17.6
Gender (m:f)	11:9
Initial cerebral pathology (n; %)	
Subarachnoid hemorrhage (SAH)	4 (20%)
Intracranial bleeding	3 (15%)
Traumatic brain injury	13 (65%)
Type of DC (n; %)	
Left hemisphere	6 (30%)
Right hemisphere	10 (50%)
Both hemispheres	4 (20%)
Timing of DC	
<24 hours	18
24 hours–72 hours	2

**Table 2:** Results from sonographic duplex technique (SDT) and cranial computed tomography (CCT) from all four ventricles.

Measured Structures	Measurement	Cranial Computed Tomography (Mean ± SD in mm)	Sonographic Duplex Technique (Mean ± SD in mm)	Pearson’s Correlation Coefficient r	Pearson’s Correlation Coefficient r for All Three Measurements	Overall Difference with 95% CI
Right lateral ventricle	1	8.1 ± 5.9	7.9 ± 5.7	0.993*		
	2	10.3 ± 5.1	9.9 ± 5.1	0.995*	0.997*	+0.3
	3	14.3 ± 13.3	13.9 ± 13.2	0.998*		[0.1–0.5]
Left lateral ventricle	1	9.3 ± 5.8	9.1 ± 5.9	0.990*		
	2	11.7 ± 5.9	11.3 ± 5.7	0.993*	0.997*	+0.4
	3	15.5 ± 12.7	15.0 ± 12.6	0.999*		[0.2–0.6]
Third ventricle	1	4.5 ± 3.2	4.3 ± 3.2	0.983*		
	2	5.7 ± 3.5	5.3 ± 3.4	0.995*	0.991*	+0.3
	3	7.4 ± 5.0	7.3 ± 4.9	0.992*		[0.1–0.4]
Fourth ventricle	1	10.7 ± 2.6	10.3 ± 2.9	0.967*		
	2	11.3 ± 2.6	10.7 ± 2.6	0.981*	0.977*	+0.5
	3	12.3 ± 2.8	11.8 ± 2.8	0.986*		[0.3–0.6]

Three measurements of mean ventricular dimensions in SDT and CCT and correlation. Data from SDT and CCT are expressed as mean ± standard deviation (SD) in mm. ^*^all p-value were < 0.001.
